# Examining sources of variability in repeated testing of the reward positivity and P300 ERP components

**DOI:** 10.3389/fnins.2026.1960493

**Published:** 2026-10-13

**Authors:** Katherine Boere, Mathew R. Hammerstrom, Jeff A. Hopkins, Isaac K. Barss, Rae Dauphinee, Patrick J. Montgomery, Olav E. Krigolson

**Affiliations:** The Theoretical and Applied Neuroscience Lab, Department of Exercise Science, Physical Health & Education, The University of Victoria, Victoria, BC, Canada

**Keywords:** EEG, electrophysiology, event-related potentials (ERP), P300, reinforcement learning, rewards positivity, test–retest reliability

## Abstract

**Introduction:**

Event-related potentials (ERPs) provide millisecond-resolution measurements of cognitive and affective processes, making them a foundational tool in human neuroscience research. A clear understanding of their reliability, stability, and sensitivity is therefore essential for interpreting experimental results with confidence, particularly in multi-task protocols where several ERPs are measured in sequence. The present study examined three sources of variability for two widely used components, the P300 and the rewards positivity: test-retest reliability, systematic amplitude changes across repeated measurements, and order effects.

**Methods:**

Twenty-six adults completed five cognitive assessments distributed over approximately 2 h, with each assessment containing a visual oddball task and a two-armed bandit task; task order was independently randomized within each assessment. Reliability was quantified using intraclass correlation coefficients (ICCs), changes across repeated assessments were tested with linear mixed-effects models, and order effects were evaluated using mixed-effects models accounting for repeated observations within participants.

**Results:**

The P300 demonstrated excellent reliability across all five assessments (ICC = 0.91). The rewards positivity showed a clear divergence between conditional and difference-wave measures: the difference wave was moderately reliable (ICC = 0.52), while the underlying win (0.92) and loss (0.92) waves demonstrated excellent reliability. Rewards positivity amplitude showed no evidence of a linear change across assessments, whereas P300 amplitude increased systematically across the testing period. No evidence of order effects was observed for either component, indicating that task order did not meaningfully influence ERP amplitude within sessions of this length.

**Discussion:**

Together, these findings characterize the reliability and time-related variability of the P300 and rewards positivity during repeated within-session testing and provide practical guidance for multi-task EEG protocols.

## 1 Introduction

In scientific endeavors, as in much of daily life, the ordering of operations is fundamental. It makes no sense to tie your running shoes before putting them on. Yet, sometimes knowing the proper order can be difficult, such as whether it is best to stretch before or after a run. In designing experiments with multiple tasks, it is often difficult to know what effect one task may have on another. In electroencephalography (EEG) research, where event-related potentials (ERPs) are measured at the microvolt scale, even small uncontrolled influences have the potential to obscure or mimic experimental effects. When ERP-eliciting tasks are administered in sequence, three sources of variability require particular attention: reliability, the consistency of a measure across repeated assessments; time-related variability, systematic changes in component amplitude across repeated assessments; and order effects, the influence of a preceding task on the ERP elicited by the following task. Each can independently alter ERP amplitudes, and failing to account for one can lead to misattribution of another.

Two ERPs central to this investigation are the rewards positivity and the P300. During rewards-based learning, the brain compares expected outcomes to actual outcomes, and the discrepancy between the two is reflected in the rewards positivity, an ERP component appearing approximately 250–350 ms after feedback at frontocentral sites (Proudfit, 2015). Larger discrepancies produce larger amplitudes, and as learning progresses and outcomes become predictable, the component diminishes (Krigolson et al., 2009; Williams et al., 2020). Converging evidence links the rewards positivity to phasic dopaminergic signaling in midbrain rewards circuits, with the signal reflected at the level of the anterior cingulate cortex (Holroyd and Coles, 2002; Proudfit, 2015). Previous work examining the test–retest reliability of the rewards positivity difference wave has reported values in the poor to moderate range, though the underlying conditional waves for win and loss feedback considered separately are substantially more reliable (Amir et al., 2023; Levinson et al., 2017; Szenczy et al., 2021).

The second notable component, the P300, peaks approximately 250–500 ms after stimulus onset and is maximal over parietal scalp sites (Polich, 2007). Functionally, the P300 has been linked to context updating, whereby the brain revises its representation of the environment in response to new information (Donchin, 1981), to attentional resource allocation during stimulus evaluation, and to neural inhibition of task-irrelevant activity in support of memory processing (Polich, 2007). Its neural basis has been traced to phasic activity of the locus coeruleus–norepinephrine system (Nieuwenhuis et al., 2005; Polich, 2007). Test–retest reliability of the P300 is generally good to excellent across delays ranging from weeks to over a year (Brunner et al., 2013; Ip et al., 2018; Kamp et al., 2023; Sandman and Patterson, 2000; Vázquez-Marrufo et al., 2013), suggesting that the component is stable enough for amplitude differences across conditions to reflect systematic influences rather than measurement noise. At the same time, P300 amplitude attenuation has been documented in sustained, cognitively demanding tasks and is typically attributed to task disengagement and mental fatigue (Hopstaken et al., 2015; Umemoto et al., 2019). However, these findings have primarily been observed during continuous task performance, where repeated stimulus exposure occurs alongside prolonged cognitive engagement. Whether comparable attenuation emerges when the same task is administered as several short assessments separated by breaks–rather than as a single continuous block–remains an open question, and one that motivates the present design. This distinction is important because separating assessments interrupts continuous task engagement while preserving repeated exposure to the ERP-eliciting paradigm. Accordingly, we examined whether P300 or rewards positivity amplitude changes across distributed assessments separated by breaks.

The present study evaluated three sources of variability in these components: test–retest reliability, within-session amplitude change, and task-order effects. Participants completed five cognitive assessments over approximately 2 h, each consisting of a visual oddball task and a two-armed bandit task, with task order independently randomized within each assessment. Reliability was assessed across all five assessments for both difference and conditional waveforms, while changes in component amplitude across assessments and task-order effects were also evaluated. Based on prior findings, we hypothesized that P300 reliability would be good to excellent and that rewards positivity difference-wave reliability would be poor to moderate, with substantially higher reliability for its underlying conditional waveforms (Levinson et al., 2017; Szenczy et al., 2021). Based on evidence of P300 attenuation during sustained cognitive engagement and reductions in rewards positivity as outcomes become increasingly predictable, we expected both components to decrease across repeated assessments (Hopstaken et al., 2015; Krigolson et al., 2009; Umemoto et al., 2019; Williams et al., 2020). Further, the temporal form of these changes during distributed repeated testing has not been established; accordingly, the linear model was used as a parsimonious test of systematic change rather than to imply that attenuation should follow a strictly linear trajectory. Finally, because no prior work speaks directly to whether sequential administration of these tasks systematically biases ERP amplitudes, our examination of order effects was exploratory and made without a directional prediction.

## 2 Materials and methods

### 2.1 Participants

Thirty-four participants were recruited from the University of Victoria and through online advertisements in Victoria, BC, as part of a larger study. Participants with incomplete datasets were excluded, resulting in a final sample of 26 participants (15 women, 10 men, 1 non-binary; mean age = 25.3 years, 95% CI [21.7, 28.9]). All participants received monetary compensation and provided informed consent in accordance with the University of Victoria Human Research Ethics Board (Protocol 21-0179) and the Declaration of Helsinki. Inclusion criteria required normal or corrected-to-normal vision.

### 2.2 Procedure

Following consent, participants were fitted with an EEG cap and seated in a sound-attenuated room approximately 60 cm from a 19-inch LCD monitor. Five cognitive assessments (CAs) were completed over approximately 2 h. CA1 served as baseline, with subsequent assessments completed at 15 (CA2), 30 (CA3), 60 (CA4), and 90 (CA5) min post-baseline (Figure 1). The timing of the five cognitive assessments was determined by the protocol of the larger parent study. Each CA consisted of a visual oddball task and a two-armed bandit task, with task order independently randomized within each cognitive assessment and therefore able to vary across assessments within the same participant. Tasks were programmed in MATLAB (R2016a; MathWorks; RRID:SCR_001622) using the Psychophysics Toolbox (Version 3.0.12; Brainard, 1997; Pelli, 1997; RRID:SCR_002881).

**FIGURE 1:**
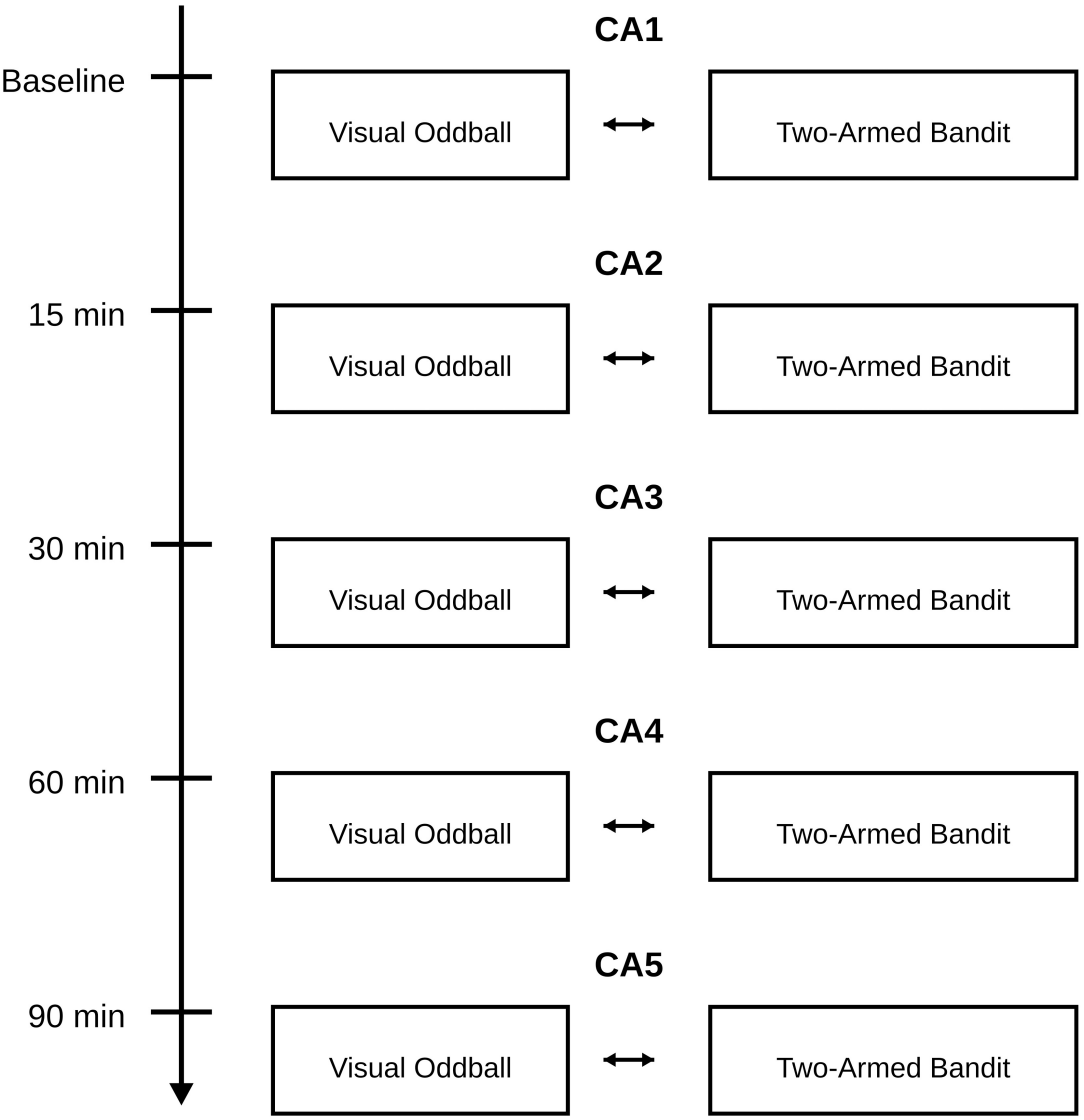
Experimental protocol schematic. Participants completed five cognitive assessments (CA1–CA5) over approximately 2 h, with assessments occurring at baseline and 15, 30, 60, and 90 min post-baseline. Each cognitive assessment included a visual oddball task and a two-armed bandit task, in an independently randomized order.

The passive visual oddball task was used to elicit the P300 (Sutton et al., 1965). Participants viewed a series of colored circles presented centrally, with green circles serving as the infrequent oddball stimulus (25%) and blue circles as the frequent stimulus (75%). No behavioral response was required. Four blocks of 50 trials were completed per assessment.

The two-armed bandit task (hereafter, the bandit task) was used to elicit the rewards positivity. On each trial, participants selected one of two colored squares after a go cue, followed by outcome feedback (“WIN” or “LOSE”). One square was associated with a higher probability of rewards (60%) than the other (10%), allowing participants to learn the advantageous option while maintaining sufficient win and loss trials. Five blocks of 20 trials were completed per assessment. Reaction time was defined as the duration from choice presentation to choice selection. Win-feedback rate was calculated as the proportion of trials resulting in WIN feedback; under random choice between the two options, the expected win-feedback rate was 35%.

### 2.3 Data acquisition and processing

Electroencephalography was recorded using a 64-electrode ActiCAP system (Brain Products GmbH, Munich, Germany) arranged according to the 10–20 layout and acquired using BrainVision Recorder (Version 1.21). Signals were sampled at 500 Hz, amplified using an ActiChamp amplifier (Revision 2), referenced to common ground during acquisition, and anti-alias low-pass filtered at 245 Hz. Stimulus presentation and event markers were synchronized using a DataPixx unit. Data were processed in MATLAB (Version 9.6; MathWorks, Natick, MA, USA) using EEGLAB (Delorme and Makeig, 2004) and custom Krigolson Laboratory scripts^1^.

Preprocessing was conducted in two passes. Initially, noisy or damaged channels were identified and interpolated using spherical splines. Data were re-referenced to the averaged mastoids (TP9 and TP10), band-pass filtered from 0.1 to 30 Hz using a fourth-order Butterworth filter, and notch filtered at 60 Hz. Ocular artifacts were corrected using independent component analysis in EEGLAB and custom functions. Data were segmented from −200 to 800 ms relative to stimulus onset, baseline corrected using the −200 to 0 ms interval, and subjected to artifact rejection using a 150-μV difference threshold and a 10% channel tolerance. A second pass was performed only for datasets exceeding the 10% artifact threshold, with preprocessing repeated after removal of affected electrodes.

Datasets with more than four removed channels or loss of any electrodes required for referencing, artifact assessment, or ERP quantification (FCz, FC2, Pz, POz, Cz, Fp1, Fp2, TP9, and TP10) were excluded. TP9 and TP10 were used for mastoid re-referencing, Fp1 and Fp2 contributed to ocular-artifact assessment, and FC2 and Pz served as the primary quantification sites for the rewards positivity and P300, respectively. The remaining electrodes included in the exclusion criteria (FCz, Cz, and POz) were retained for data-quality assessment and topographic evaluation. Participants were retained only if all five cognitive assessments met these criteria (23% excluded, resulting in a final dataset of *n* = 26). Across all data, the mean artifact-rejection rate was 4.0% ± 4.7% (range: 0%–22.2%). Retained trial counts for each condition and cognitive assessment are reported in Supplementary Table 1 as mean, standard deviation, and range.

For the oddball task, frequent and oddball trials were averaged at Pz, with the P300 defined as the difference between oddball and frequent waveforms. Pz was selected *a priori* as the P300 quantification site based on the characteristic parietal distribution of the P3b and its conventional use in oddball paradigms (Polich, 2007). For the rewards task, win and loss trials were averaged at FC2, which was identified from the grand-average scalp distribution as the site of maximal rewards positivity amplitude in the present dataset. This frontocentral distribution is consistent with previous methodological and large-sample work characterizing the rewards positivity (Williams et al., 2021). The rewards positivity was defined as the difference between win and loss waveforms.

Amplitudes were quantified using mean peak measures (Luck, 2014; Williams et al., 2021). Window sizes of ±54 ms for the P300 and ±46 ms for the rewards positivity were determined as the inflection point of mean amplitudes across a series of window sizes from 0 to 100 ms (Williams et al., 2021). For the P300, amplitude was calculated as the mean voltage within ±54 ms of the grand average peak latency (366 ms). For the rewards positivity, each participant’s local peak was identified as the maximum of their difference wave within ±46 ms of the grand average peak latency (304 ms), and amplitude was calculated as the mean voltage within ±46 ms of this local peak. Where no local peak was identifiable, the grand average latency was used.

### 2.4 Data analysis and statistics

All statistical analyses were performed using R (Version 4.2.1; R Foundation for Statistical Computing, Vienna, Austria) and RStudio (Version 2022.7.2.576; RStudio Team, Boston, MA, USA). Figures were created using GraphPad Prism (Version 10.2.3; RRID:SCR_002798).

Test–retest reliability was assessed using intraclass correlation coefficients (ICCs) for both components. A two-way random-effects, absolute-agreement, multiple-measurement model [ICC(2, *k*)] was selected according to the guidelines of Koo and Li (2016). Reliability was classified as poor (<0.50), moderate (0.50–0.75), good (0.75–0.90), or excellent (>0.90) based on ICC values (Koo and Li, 2016). Given prior reports of lower reliability for rewards positivity difference waves and higher reliability for the underlying conditional measures, ICCs were also calculated separately for the win and loss waveforms (Levinson et al., 2017; Szenczy et al., 2021). For consistency, conditional ICCs were likewise calculated separately for the oddball and frequent P300 waveforms.

Changes in component amplitude across CAs were assessed using linear mixed-effects models. Participant was included as a random intercept, and CA number was entered as a continuous fixed effect. This approach tested linear change across assessments while accounting for between-participant differences in component amplitude. Planned comparisons were conducted using paired-sample *t*-tests comparing CA1 with CA2 and CA1 with CA5.

To examine order effects, rewards positivity and P300 amplitudes were classified according to whether the corresponding task was completed first or second within each CA. Task order was independently randomized within each assessment, resulting in 70 oddball-first and 60 bandit-first observations across the 130 CA-level observations. Specifically, the oddball-first/bandit-first allocation was 15/11 at CA1, 13/13 at CA2, 19/7 at CA3, 13/13 at CA4, and 10/16 at CA5. Overall order effects were assessed using linear mixed-effects models with task order and CA entered as fixed effects and participant included as a random intercept. CA was treated as a categorical factor to account for differences across assessments without imposing a linear trend. To determine whether the effect of task order varied across assessments, a second model including an Order × CA interaction was compared with the corresponding main-effects model using a likelihood-ratio test.

## 3 Results

### 3.1 Test–retest reliability

Test–retest reliability was assessed using ICC(2, k) for P300 and rewards positivity amplitudes. P300 reliability was excellent, ICC = 0.91, *p* < 0.001, as was reliability for the oddball, ICC = 0.91, *p* < 0.001, and frequent, ICC = 0.93, *p* < 0.001, conditional waveforms. Rewards positivity reliability was moderate, ICC = 0.52, *p* = 0.006. Reliability of the conditional win and loss waveforms was excellent (win: ICC = 0.92, *p* < 0.001; loss: ICC = 0.92, *p* < 0.001).

### 3.2 Amplitude changes and order effects

Event-related potential difference waveforms across CA1–CA5 are shown in Figures 2A,C, with conditional waveforms shown in Figures 3A–J. Behavioral performance on the two-armed bandit task across CA1–CA5 is presented in Supplementary Table 2.

**FIGURE 2:**
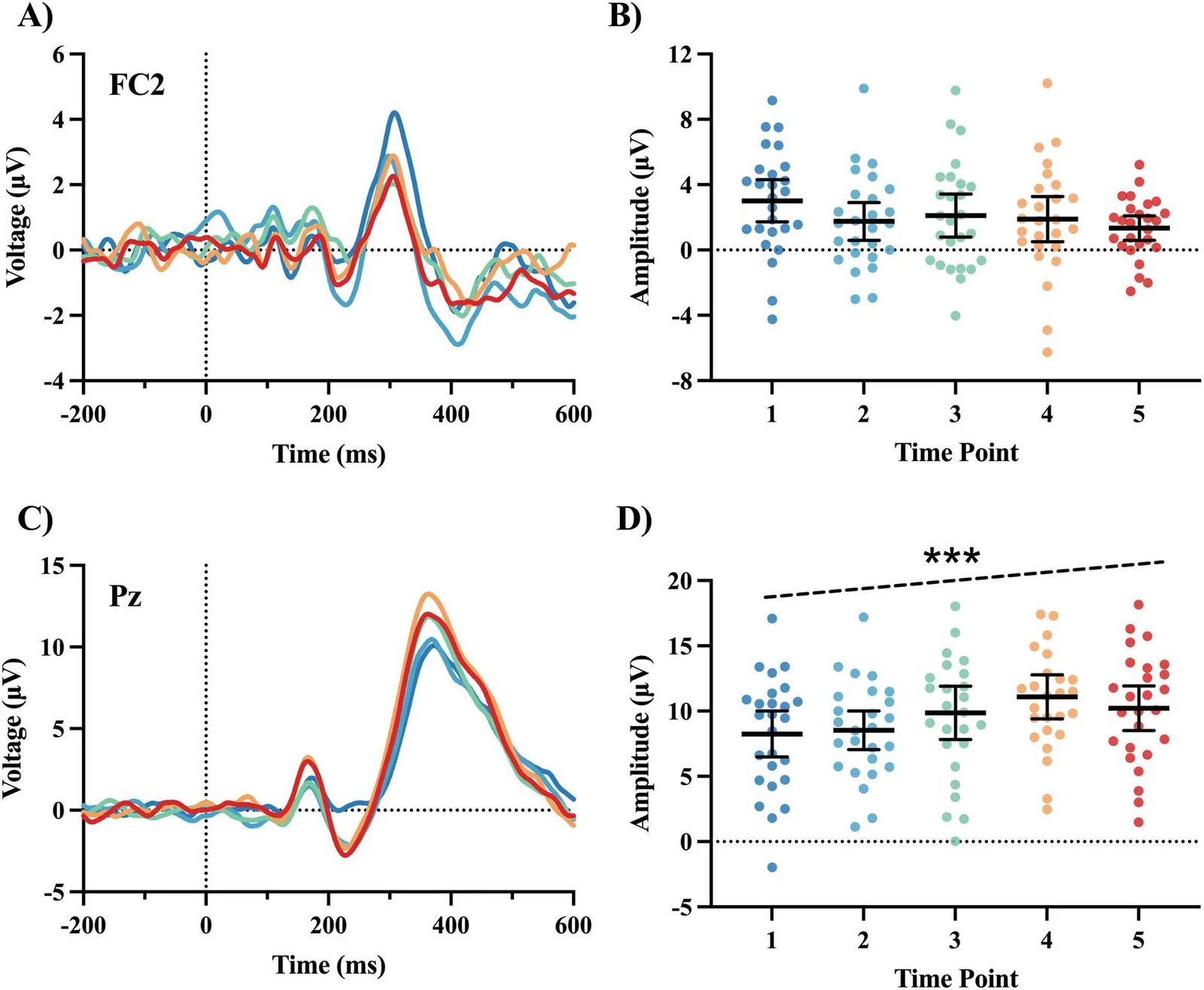
Grand-average ERP difference waveforms and amplitude trajectories across five cognitive assessments. **(A)** Rewards positivity at FC2 and **(C)** P300 at Pz across CA1–CA5. **(B,D)** Mean peak amplitudes across assessments. Points represent individual participants; bars indicate mean ± 95% CI. The dashed line in panel **(D)** represents the linear mixed-effects model estimate. ***p < 0.001.

**FIGURE 3:**
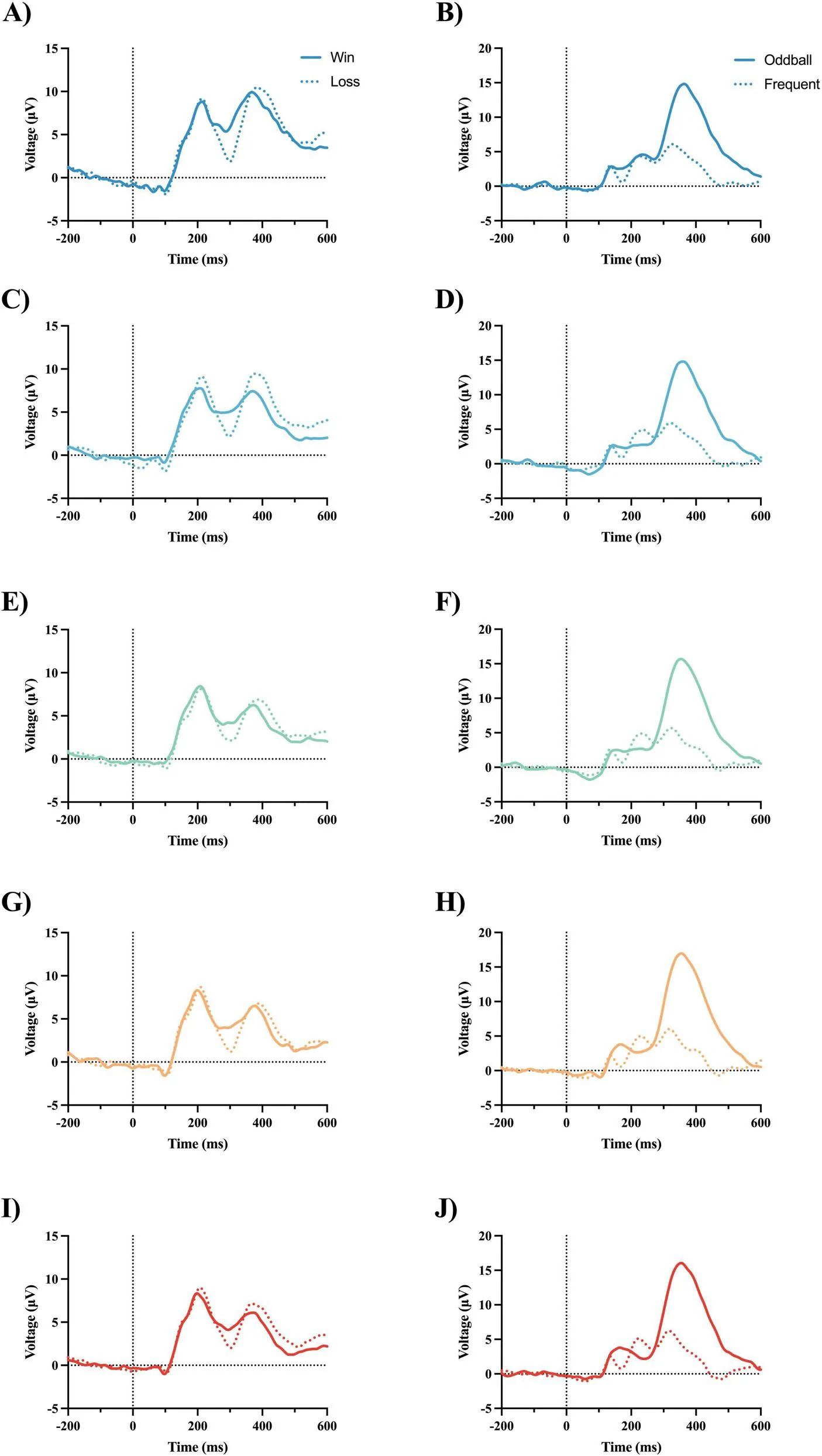
Grand-average conditional ERP waveforms across five cognitive assessments. **(A,C,E,G,I)** Show win and loss waveforms for the rewards positivity at FC2 across CA1–CA5, respectively. **(B,D,F,H,J)** Show oddball and frequent waveforms for the P300 at Pz across CA1–CA5, respectively. Solid and dotted lines represent the respective task conditions.

Changes in component amplitude across CAs were assessed using linear mixed-effects models, with CA number included as a fixed effect and participant included as a random intercept. Likelihood-ratio tests were used to evaluate the contribution of CA to each model.

For the rewards positivity (Figure 2B), there was no evidence of a linear effect of CA on component amplitude, χ^2^(1) = 3.69, *p* = 0.055, with a slope of −0.32 ± 0.17 μV per assessment, 95% CI [−0.65, 0.01] (random-effects SD = 1.27; residual SD = 2.68). Planned comparisons showed no difference between CA1 and CA2, MD = 1.23 μV, *t*(25) = 1.64, *p* = 0.113, but a difference between CA1 and CA5, MD = 1.67 μV, *t*(25) = 2.97, *p* = 0.006.

For the P300 (Figure 2D), CA affected component amplitude, χ^2^(1) = 18.50, *p* < 0.001, with a positive slope of 0.65 ± 0.15 μV per assessment, 95% CI [0.36, 0.94] (random-effects SD = 3.67; residual SD = 2.34). Planned comparisons showed no difference between CA1 and CA2, MD = −0.28 μV, *t*(25) = −0.40, *p* = 0.696, but a difference between CA1 and CA5, MD = −1.97 μV, *t*(25) = −3.23, *p* = 0.003.

Task order was examined using linear mixed-effects models that accounted for repeated observations within participants and differences across CAs. There was no evidence of an overall effect of task order on either rewards positivity amplitude, χ^2^(1) = 0.13, *p* = 0.719, or P300 amplitude, χ^2^(1) = 0.69, *p* = 0.406. Adding an Order × CA interaction did not improve model fit for either the rewards positivity, χ^2^(4) = 3.72, *p* = 0.445, or the P300, χ^2^(4) = 6.35, *p* = 0.175.

## 4 Discussion

The present study examined three sources of variability that can shape ERP amplitudes when the P300 and the rewards positivity are measured repeatedly within a single session: test–retest reliability, changes in amplitude across assessments, and task-order effects. Both components demonstrated reliable measurement, although the pattern differed by waveform type. The rewards positivity difference wave showed moderate reliability, while its conditional waveforms demonstrated excellent reliability, whereas P300 reliability was excellent across all measures. Regarding amplitude trajectories, P300 amplitude increased linearly across assessments, whereas rewards positivity amplitude showed no evidence of a linear change. No evidence of an effect of task order was detected for either component.

Reliability of the P300 was excellent across the five repeated assessments (ICC = 0.91), with comparable values for both the oddball (ICC = 0.91) and frequent (ICC = 0.93) conditional waveforms. Prior work has reported good-to-excellent test–retest reliability of the P300 across delays ranging from weeks to over a year (Brunner et al., 2013; Ip et al., 2018; Kamp et al., 2023; Sandman and Patterson, 2000). The present findings extend this work by demonstrating similarly high reliability across repeated assessments conducted within a single session, suggesting that amplitude differences observed across assessments are unlikely to reflect measurement noise alone.

Reliability of the rewards positivity was more nuanced. Whereas the difference wave produced a moderate ICC (0.52), the conditional win (0.92) and loss (0.92) waveforms both demonstrated excellent reliability. This pattern is consistent with previous reports showing substantially greater reliability for the constituent feedback responses than for their difference (Amir et al., 2023; Levinson et al., 2017; Szenczy et al., 2021). Lower reliability of a difference score can arise when its constituent measures are highly correlated, because subtraction removes variance shared across the two conditions while retaining condition-specific and error variance. Thus, high reliability of the conditional win and loss waveforms can coexist with lower reliability of their difference. For studies relying on the rewards positivity as an individual-difference measure, reporting the underlying conditional waveforms alongside the difference score may therefore provide useful additional information about measurement reliability.

P300 amplitude increased linearly across the five assessments, the inverse of the attenuation documented in sustained, fatigue-inducing paradigms (Hopstaken et al., 2015; Umemoto et al., 2019). Several mechanisms may account for this unexpected pattern. Repeated exposure to the task may have improved the efficiency of stimulus evaluation and probability estimation, reducing the resources required for these processes and allowing greater allocation to the context-updating operations associated with the P3b (Donchin and Coles, 1988; Lindín et al., 2004; Polich, 2007). The distributed structure of the protocol may also have contributed, as interblock intervals have previously been shown to influence P300 amplitude during repeated testing (Wesensten et al., 1990). However, because the present study did not include a continuous no-break comparison condition, the contribution of the intervening rest periods cannot be isolated. Future work manipulating task exposure and inter-assessment interval independently will be needed to distinguish these possibilities. Regardless of mechanism, the present findings indicate that P300 amplitude can change systematically across repeated short assessments and should therefore be considered when interpreting repeated-measures ERP designs.

Rewards positivity amplitude did not show evidence of a linear change across the session. The planned CA1 versus CA2 comparison was also not significant, although the CA1 versus CA5 contrast differed (*p* = 0.006). One possible explanation for the CA1–CA5 difference is increased familiarity with the rewards contingencies over the course of the protocol; as outcomes become more predictable, the prediction-error signal driving the rewards positivity may diminish (Krigolson et al., 2009; Williams et al., 2020). However, the absence of a linear effect suggests that any change in rewards positivity was not progressive across assessments. Further work incorporating direct measures of learning across repeated assessments would help clarify whether changes in rewards expectancy contribute to within-session variation in rewards positivity amplitude.

No evidence of order effects was detected for either component. Mixed-effects models showed no overall effect of task order on rewards positivity or P300 amplitude, and the Order × CA interaction did not improve model fit for either component. Because the P300 and rewards positivity have been associated with partly distinct neuromodulatory systems–noradrenergic locus coeruleus activity for the former and dopaminergic signaling associated with anterior cingulate cortex function for the latter–it was plausible that prior engagement of one system could influence the subsequent response of the other. However, the present data did not support this possibility. Under the current protocol, sequential administration of these two tasks therefore did not produce a detectable systematic bias in ERP amplitude across the testing session.

Several limitations warrant consideration. Modest sample size (*n* = 26) constrains power to detect small order effects, and a single 2-h session does not address test–retest reliability across the days-to-weeks intervals most relevant to clinical and longitudinal applications. The timing of the repeated assessments also limits interpretation of the observed amplitude trajectories. Inter-assessment intervals were unequal (15, 15, 30, and 30 min), such that assessment number was confounded with the duration of the intervening interval. Consequently, effects associated with repeated task exposure cannot be fully separated from those associated with elapsed time or differing opportunities for recovery. Only one task pair was examined, and generalization to ERP combinations engaging more overlapping neuromodulatory systems remains untested; future work systematically varying task pairing would clarify the boundary conditions of the present order-effect findings. Subjective fatigue and motivation were not assessed directly, which limits the ability to adjudicate between practice and fatigue as accounts of the P300 trajectory; incorporating self-report measures of fatigue and engagement across assessments would allow these possibilities to be examined more directly. Future work should also replicate these findings in more diverse samples and extend repeated assessments across sessions separated by longer delays to address the reliability timescales most relevant to applied ERP research.

## 5 Conclusion

The present study characterized the reliability, within-session amplitude changes, and order effects of the P300 and rewards positivity across five short cognitive assessments conducted within a single 2-h session. P300 reliability was excellent, whereas rewards positivity reliability was moderate for the difference wave but excellent for the underlying win and loss waveforms. Across the session, P300 amplitude increased systematically, whereas rewards positivity amplitude showed no evidence of a linear change. No detectable effect of task order was observed for either component. These findings support reporting conditional waveforms alongside rewards positivity difference scores and highlight the importance of considering time-related amplitude changes when the oddball P300 is measured repeatedly within a session.

## Data Availability

The datasets presented in this study can be found in online repositories. The names of the repository/repositories and accession number(s) can be found below: https://osf.io/k8f3e/overview?view_only=0081932532f14c68bfc25abef9c6fdfa.

## Footnotes

1
https://github.com/krigolson
