# Supplementary material for: Examining sources of variability in repeated testing of the reward positivity and P300 ERP components
Source: Front Neurosci. 2026 Oct 13;20:1960493. doi: 10.3389/fnins.2026.1960493 (PMC13729249; doi:10.3389/fnins.2026.1960493)
Supplement: Supplementary file 1 [file Table_1.docx]

**Supplementary Table S1.** Retained trial counts after artifact rejection for each condition across cognitive assessments.

| **Cognitive assessment** | **Win** | **Loss** | **Oddball** | **Frequent** |
| --- | --- | --- | --- | --- |
| CA1 | 43.7 ± 7.0 [32–60] | 42.2 ± 9.8 [18–61] | 48.1 ± 8.5 [32–63] | 138.1 ± 10.1 [108–152] |
| CA2 | 47.6 ± 9.7 [25–59] | 44.5 ± 7.0 [30–58] | 48.4 ± 5.9 [35–58] | 145.3 ± 8.7 [123–165] |
| CA3 | 50.4 ± 6.7 [35–60] | 44.1 ± 6.8 [29–58] | 47.8 ± 5.5 [38–58] | 147.3 ± 8.0 [128–159] |
| CA4 | 50.4 ± 5.7 [36–59] | 45.3 ± 6.0 [33–58] | 46.8 ± 6.6 [34–62] | 149.7 ± 7.7 [132–165] |
| CA5 | 48.9 ± 8.6 [27–64] | 45.2 ± 7.1 [28–57] | 50.3 ± 5.3 [42–67] | 146.8 ± 7.0 [129–156] |

*Values are mean ± SD [range].*

**Supplementary Table S2.** Behavioral performance on the two-armed bandit task across cognitive assessments.

| **Cognitive assessment** | **Reaction time (ms), mean ± SD** | **Win-feedback rate (%), mean ± SD** |
| --- | --- | --- |
| CA1 | 376.6 ± 173.1 | 51.3 ± 7.3 |
| CA2 | 318.6 ± 144.3 | 51.6 ± 7.1 |
| CA3 | 303.8 ± 131.2 | 53.1 ± 6.0 |
| CA4 | 296.7 ± 129.7 | 52.5 ± 5.9 |
| CA5 | 293.4 ± 144.6 | 51.9 ± 6.7 |

*Values are mean ± SD.*
